# Safety and Efficacy of DT-DEC01 Therapy in Duchenne Muscular Dystrophy Patients: A 12 - Month Follow-Up Study After Systemic Intraosseous Administration

**DOI:** 10.1007/s12015-023-10620-3

**Published:** 2023-09-14

**Authors:** Maria Siemionow, Grzegorz Biegański, Adam Niezgoda, Jacek Wachowiak, Jarosław Czarnota, Krzysztof Siemionow, Anna Ziemiecka, Maria H. Sikorska, Katarzyna Bożyk, Ahlke Heydemann

**Affiliations:** 1 Chair and Department of Traumatology, Orthopedics and Surgery of the Hand, Poznan University of Medical Sciences, 61‑545 Poznan, Poland; 2 Dystrogen Therapeutics Corp., Chicago, IL 60609 USA; 3 Department of Orthopaedics, University of Illinois at Chicago, Chicago, IL 60612 USA; 4 Department of Infectious Diseases and Child Neurology, Poznan University of Medical Sciences, 60‑572 Poznan, Poland; 5 Department of Neurology, Poznan University of Medical Sciences, 60-355 Poznan, Poland; 6 Department of Pediatric Oncology, Hematology and Transplantology, Poznan University of Medical Sciences, 60-572 Poznan, Poland; 7 Hospital MedPolonia, 60-693 Poznan, Poland; 8 Department of Physiology and Biophysics, University of Illinois at Chicago, Chicago, IL 60612 USA; 9 Center for Cardiovascular Research, University of Illinois at Chicago, Chicago, IL 60612 USA

**Keywords:** Duchenne Muscular Dystrophy, Dystrophin Expressing Chimeric (DEC) Cell, Electromyography (EMG), Safety, Stem Cell Therapy

## Abstract

Duchenne Muscular Dystrophy (DMD) is a progressive and fatal muscle-wasting disease with no known cure. We previously reported the preliminary safety and efficacy up to six months after the administration of DT-DEC01, a novel Dystrophin Expressing Chimeric (DEC) cell therapy created by fusion of myoblasts of DMD patient and the normal donor. In this 12-month follow-up study, we report on the safety and functional outcomes of three DMD patients after the systemic intraosseous administration of DT-DEC01. The safety of DT-DEC01 was confirmed by the absence of Adverse Events (AE) and Severe Adverse Events (SAE) up to 21 months after intraosseous DT-DEC01 administration. The lack of presence of anti-HLA antibodies and Donors Specific Antibodies (DSA) further confirmed DT-DEC01 therapy safety. Functional assessments in ambulatory patients revealed improvements in 6-Minute Walk Test (6MWT) and timed functions of North Star Ambulatory Assessment (NSAA). Additionally, improvements in PUL2.0 test and grip strength correlated with increased Motor Unit Potentials (MUP) duration recorded by Electromyography (EMG) in both ambulatory and non-ambulatory patients. DT-DEC01 systemic effect was confirmed by improved cardiac and pulmonary parameters and daily activity recordings. This follow-up study confirmed the safety and preliminary efficacy of DT-DEC01 therapy in DMD-affected patients up to 12 months after intraosseous administration. DT-DEC01 introduces a novel concept of personalized myoblast-based cellular therapy that is irrespective of the mutation type, does not require immunosuppression or the use of viral vectors, and carries no risk of off target mutations. This establishes DT-DEC01 as a promising and universally effective treatment option for all DMD patients.

**Graphical Abstract:**

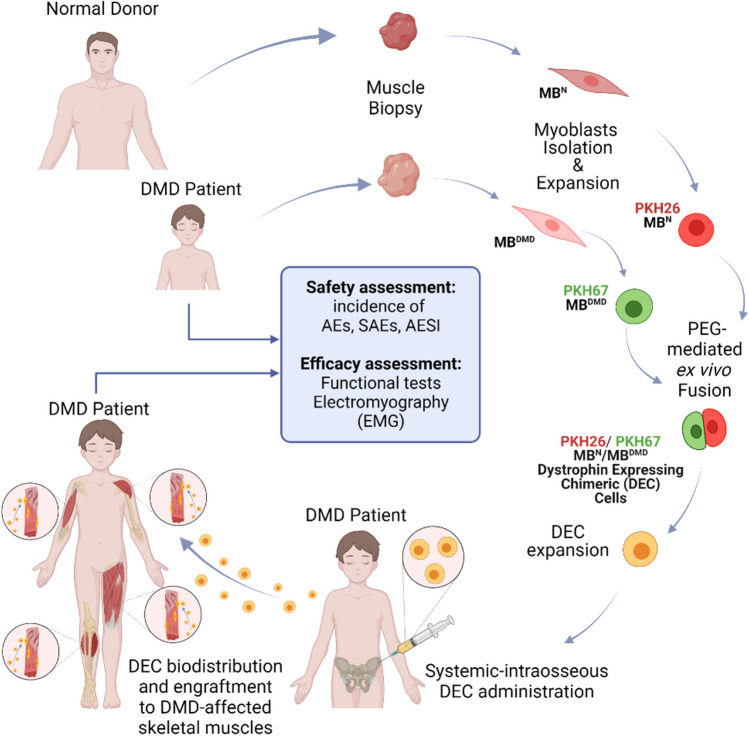

## Introduction

Duchenne muscular dystrophy (DMD) is a severe, progressive and lethal X-linked disease caused by mutations in the dystrophin gene that affects 1 in 3600–6000 live male births [1]. Absence of dystrophin is responsible for progressive muscle weakness affecting both cardiac and skeletal muscles, leading to premature death due to cardiopulmonary complications.

Currently, there is no specific approved treatment that could halt or reverse progression of DMD.

The supportive measures to alleviate DMD symptoms include corticosteroids [1], beta-blockers or angiotensin receptor blockers often given prophylactically [2] and with progression of the disease, nighttime assisted ventilation is added during adolescence [3]. These strategies have reduced DMD progression and improved the quality of life of DMD patients, however none of the approaches lead to significant extension of the life expectancy or reduced progression of cardiomyopathy or pulmonary failure [4–6].

There are several disease modifying strategies such as exon skipping therapies or microdystrophin based options, however these approaches are limited to the selected population of patients which are fitting into the specific exon skipping therapy or population of patients which would be not sensitized to the viral vectors used for therapy manufacturing [7–17].

Therefore, there is an urgent need for novel therapeutic strategies with a broader, preferentially universal approach to address these challenges and make therapies available to all DMD patients regardless of gene mutation, disease progression or the ambulatory status.

One novel approach includes transplantation of muscle stem cells which was considered historically as a natural source of cells and showed early encouraging outcomes [18–20]. However, challenges included limited efficacy in the long-term myoblast engraftment, the need for immunosuppression to avoid cell rejection and limited evidence of significant functional improvements [18, 21–27].

To address these challenges, we have developed the novel, myoblast-based therapy of Dystrophin Expressing Chimeric (DEC) cells created via PEG-mediated ex vivo fusion of myoblasts from normal and DMD-affected donors [28–34]. Preclinical in vitro studies confirmed that DEC cells displayed phenotype and genotype of the parent cells, expressed dystrophin and maintained proliferative and myogenic differentiation potential [29]. Moreover, tolerogenic and immunomodulatory properties of DEC facilitated long-term engraftment correlating with increased dystrophin expression improvement of functional outcomes of cardiac, respiratory and skeletal muscles with no evidence of adverse side effects and no need for immunosuppression.

Moreover, to address the limited cell engraftment and lack of systemic effect after local-intramuscular injections [18–20, 28, 29, 31], based on our preclinical studies, we have introduced intraosseous administration of DEC to provide global and systemic delivery of cells to all DMD-affected organs. In the preclinical studies, the systemic effect of intraosseous DEC administration was confirmed by the long-term amelioration of the cardiac, pulmonary and skeletal muscle function which correlated with increased dystrophin expression, improved muscle morphology and reduced inflammation, fibrosis and overall mdx pathology [30–34]. These findings introduced DEC as a promising and novel therapeutic approach for patients with Duchenne Muscular Dystrophy, and encouraged the initiation of the first-in-human pilot study [35].

Recently, we confirmed safety and preliminary efficacy of DT-DEC01 therapy up to six months after systemic-intraosseous administration of DT-DEC01 in the first three DMD patients enrolled in the study [35]. To further assess safety and maintenance of preliminary efficacy observed during the first six months after intraosseous DEC administration, here we confirm the safety up to 21 months and provide summary of functional efficacy assessed up to 12 months following systemic-intraosseous administration of DT-DEC01 therapy.

## Methods

### Study Design and Participants

This pilot single-site, open-label study was initiated on August 26, 2021, to assess the safety and efficacy of a systemic-intraosseous administration of a single dose of DT-DEC01 therapy, as previously reported [35]. The study was approved by the Bioethics Committee at the Regional Medical Council in Poznan, Poland (approval no. 46/2019) and was conducted in accordance with the Good Clinical Practice (GCP) guidelines and the Declaration of Helsinki. An independent Data and Safety Monitoring Board provided safety oversight. The participants and the donors were enrolled according to the inclusion and exclusion criteria of the study protocol. Written informed consent was obtained from the myoblast donors, participants' parents or legal guardians, and participants over 13 years old. The study enrolled three male DMD patients, of age 6–15 years old, with genetically confirmed DMD, irrespective of mutation type and ambulatory status (ambulatory *n* = 2, non-ambulatory *n* = 1). Participants and donors who were enrolled in the study underwent screening to assess their medical history, physical examination, and serology. Subsequently, they underwent a muscle tissue biopsy, followed by systemic-intraosseous administration of DT-DEC01 therapy to the DMD patients at a dosage of 2 × 10^6^ cells/kg.

**Fig. 1:**
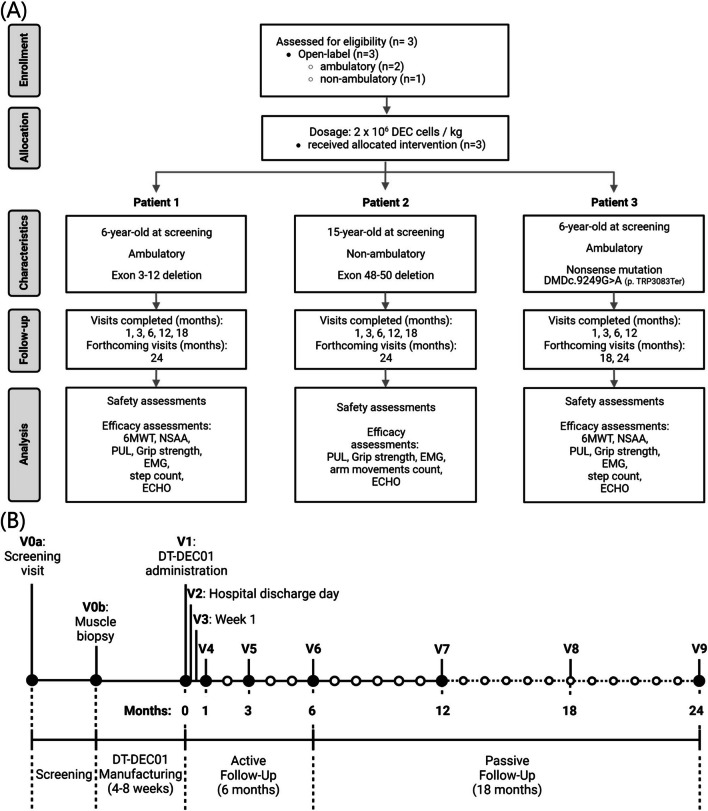
Outline of the first-in-human pilot study assessing safety and efficacy of systemic-intraosseous administration of DT-DEC01 therapy in DMD patients. **A** Consort Diagram. Enrollment: Male DMD patients of age 5–18 years old were screened and enrolled in the single-center, nonrandomized, open-label interventional study approved by the Bioethics Committee (approval no. 46/2019). Allocation: First three DMD patients who met the inclusion criteria, were assigned to receive DT-DEC01 therapy at a single dose of 2 × 10^6^ cells/kg body weight. Characteristics: Baseline demographic data, including age, ambulatory status (Patient 1 and Patient 3 were ambulatory, while Patient 2 was non-ambulatory), and DMD mutation, were collected during the screening visit (V0a). Follow-up: The total study duration is 24 months, and as of August 11, 2023, all three patients completed follow-up visits at 1, 3, 6, and 12 months after intraosseous DT-DEC01 administration. Patient 1 and Patient 2 also completed the 18-month visit. Analysis: Safety outcomes measures, assessed through the incidence of Adverse Events (AEs), Serious Adverse Events (SAEs), Adverse Events of Special Interest (AESIs), and the presence of anti-HLA antibodies, as well as efficacy outcomes measures, including functional assessments adjusted to the stage of the disease (6MWT, NSAA, PUL, grip strength, EMG, step count, ECHO for ambulatory patients; PUL, grip strength, EMG, arm movement count, ECHO for non-ambulatory patients), were collected and analyzed. **B** The patients' visit schedule included screening (V0a), skeletal muscle biopsy of DMD patient and normal donor (V0b), intraosseous DT-DEC01 administration (V1), active 6-month follow-up visits: Visit 2—Hospital Discharge Day; V3 – Week 1; V4 – Month 1; V5 – Month 3; V6—Month 6; passive 18-month follow-up visits: V7 – Month 12, V8 – Month 18, V9 – Month 24). Figure created with BioRender.com

The Study Flow Diagram is outlined in Fig. 1A and the patients visits schedule is outlined in Fig. 1B.

### Manufacturing of the Personalized DT-DEC01 Therapy Product

**Fig. 2:**
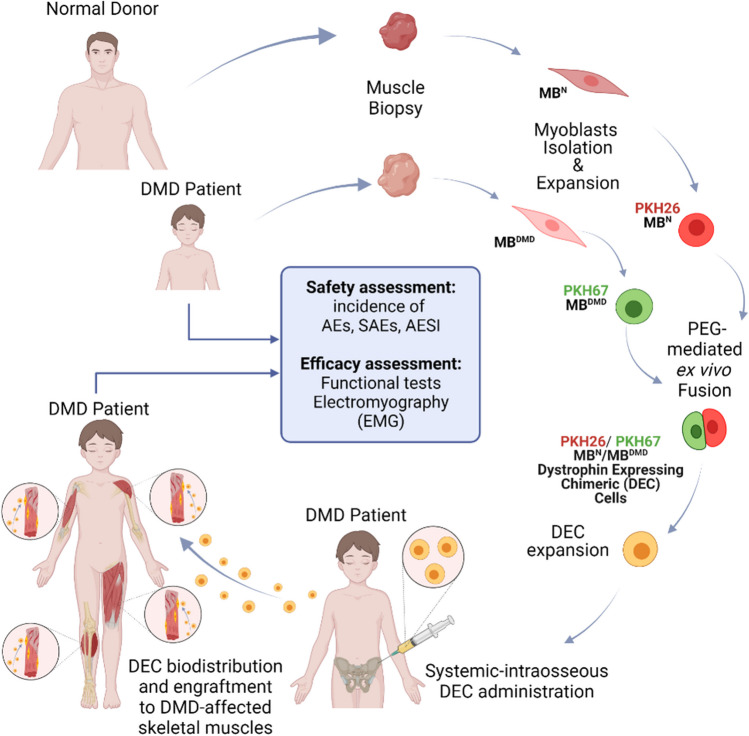
Outline of the first-in-human pilot study and manufacturing process of DT-DEC01 therapy for the assessment of safety and efficacy after systemic intraosseous administration of DT-DEC01 in DMD patients. Manufacturing of DT-DEC01 begins with muscle biopsies harvested from the DMD patient and the normal donor, followed by myoblasts isolation and expansion, PKH staining and PEG-mediated cell fusion creating Dystrophin Expressing Chimeric (DEC) cells, followed by DEC sorting, expansion, product formulation and DT-DEC01 administration to DMD patient. Figure created with BioRender.com

The established protocol for myoblast cell isolation, fusion, and DT-DEC01 manufacturing was followed as previously described and is outlined on Fig. 2 [35, 36]. Briefly, following muscle biopsy, the muscle tissue samples were digested with collagenase to isolate myoblasts. The myoblasts were propagated and passaged to reach the desired cell number for the fusion procedure. Fluorescent labeling of normal donor and DMD patient myoblast cells was followed by ex-vivo cell fusion procedure using PEG. Following fusion, the double-positive (PKH26/PKH67) chimeric DEC cells were then selected using FACS MACSQuant Tyto sorter. The manufacturing process continued to achieve the personalized dose of 2 × 10^6^ cells per kg body weight for systemic-intraosseous administration to the DMD patients.

### Systemic-intraosseous DT-DEC01 Administration

The DMD-affected patients received DT-DEC01 product via intraosseous administration to the bone marrow compartment of the patient's iliac crest, as previously described [35]. The procedure, conducted under anesthesia, included bone marrow aspiration to create space, followed by injection of a personalized dose of 2 × 10^6^ DEC cells per kg body weight into the marrow cavity, and ensuring appropriate closure and protection of the surgical site. On average, the intraosseous DT-DEC01 administration procedure lasted 7 min. Following administration, patients were hospitalized for 24 h and closely monitored for any potential responses associated with the procedure.

### DT-DEC01 Therapy Safety Assessments

The primary focus of this Pilot study was to evaluate safety by assessing the occurrence and severity of all treatment-related Adverse Events (AE), Serious Adverse Events (SAE), and Adverse Events of Special Interest (AESI). Safety evaluation was monitored from the muscle tissue biopsy on visit V0b, through the DT-DEC01 therapy administration (visit V1), followed by the active 6-month follow-up (visits V2—Hospital Discharge Day; V3 – Week 1; V4 – Month 1; V5 – Month 3; V6—Month 6), and was continued for 6 months of passive-follow up to the one-year interim study endpoint (visit V7 – Month 12). The passive follow up was continued for additional 12 months (visits V8 – Month 18, V9 – Month 24) for the total of 24 months observation after DT-DEC01 therapy administration at the study endpoint (V9). Patients were carefully and continuously monitored for any changes in the vital signs, physical exam, and laboratory tests. Assessment of any signs of local and/or systemic intra- and post-infusion complications were recorded. Furthermore, patients were screened for the presence of anti-HLA antibodies and the Donor Specific Antibodies (DSA) at 1, 3, 6 and 12 months after systemic DT-DEC01 administration, as previously reported [35].

#### Assessment of the anti-HLA antibodies and Donor Specific Antibodies (DSA)

At the screening visit (V0a) DMD patients' sera were tested for the presence of anti-HLA class I, anti-HLA class II, and anti-MICA IgG antibodies using LABScreen Mixed on the Luminex platform. In the case of a positive result, further analysis was employed to determine the presence of the pre-existing DSA (LABScreen Single Antigen HLA Class I and Class II, One Lambda, Luminex platform). If DSA was detected in a DMD patient, the donor was excluded, and another donor was screened for allogeneic myoblast donation. To assess the potential immune response to DT-DEC01 therapy, anti-HLA testing was assessed during follow-up visits at 1, 3, 6, and 12 months after systemic-intraosseous DT-DEC01 administration.

### Assessment of DT-DEC01 Therapy Efficacy by Functional Tests

Efficacy was evaluated using standardized functional tests adjusted to the disease stage. In ambulatory patients assessment included 6-Minute Walk Test (6MWT) and timed tests of NorthStar Ambulatory Assessment (NSAA). Both ambulatory and non-ambulatory patients were assessed by the Performance of Upper Limb test (PUL 2.0) and measurements of grip strength of both hands by dynamometer. These tests were conducted during the screening visit (V0a) and during follow-up visits at 1, 3, 6, and 12 months (V4, V5, V6, and V7) for comparative analysis of outcomes after systemic-intraosseous administration of DT-DEC01 therapy.

Electromyography (EMG) parameters of Motor Units Potentials (MUP) were recorded in both, ambulatory and non-ambulatory patients in the selected muscles of the upper and lower extremities at baseline (V0a) and at 3, 6, and 12 months (V5, V6, and V7) after DT-DEC01 administration.

Echocardiography (ECHO) monitored cardiac function in all patients during all follow-up visits after DT-DEC01 administration. Spirometry was assessed in the older, non-ambulatory patient. All these tests were performed following standardized methods and in appropriate conditions, ensuring patient safety. Additionally, monitoring of patients’ daily activity was assessed by the step or arm movement count recorded by wristband activity tracker (Vívosmart 4, Garmin).

#### The 6-Minute Walk Test (6MWT)

The 6MWT was performed in ambulatory patients, indoors on the flat, non-slippery surface with marked cones indicating a 30-m walking path. Patients were instructed to complete laps within 6 min at their normal pace, with the option to rest or use gait aids. The total distance covered was calculated by counting complete 30-m walks and any remaining partial laps.

#### Timed Functions of North Star Ambulatory Assessment (NSAA)

Ambulatory patients performed NSAA activities [37, 38] to assess their physical functioning, which included two timed functional tests: time to stand from supine and time to run or walk 10 m. The duration required to complete these tasks was recorded in seconds using a stopwatch, while observations were made regarding compensatory movements, particularly the presence of Gowers's sign during the process of standing from a supine position.

#### Performance of Upper Limb (PUL 2.0)

Both ambulatory and non-ambulatory patients participated in the Performance of Upper Limb assessment to evaluate their upper limb function at high (shoulder), mid (elbow), and distal (wrist and hand) levels as described in detail before [35]. The assessment was conducted using the PUL 2.0 scoresheet proposed by Pane et al. 2018 [39, 40].

#### Hand Grip Strength

Grip strength of both the right and left hand was measured using a handheld electronic dynamometer (WWEH101, Moga). Patients were seated and instructed to bend their elbow at a 90° angle and perform three consecutive voluntary contractions of each hand, applying maximum force while holding the dynamometer. Grip strength was recorded in kilograms.

#### Electromyography (EMG)

Electromyography evaluation was performed following standard procedures by a certified neurologist with expertise in assessing DMD patients. Briefly, concentric needle electrodes (Neuroline Concentric, Ambu, 28G, 30 mm) were inserted into selected muscles of the upper right extremity (deltoideus and biceps brachii) and lower right extremity (rectus femoris and gastrocnemius). The VIASYS Synergy EMG System (Medelec) automatically recorded a minimum of 10 Motor Unit Potentials (MUPs) from each muscle during mild and submaximal voluntary contractions for a total of over 160 MUPs per patient The recorded MUPs were assessed for the duration, amplitudes and the percentage of polyphasic MUP and were quantitatively analyzed using the Synergy application software (Viasys Synergy, Medelec).

#### Echocardiography (ECHO)

Echocardiography (ECHO) was performed using a GE Healthcare Vivid T8 ultrasound system. A transducer probe was used to capture real-time images of the heart by scanning across the chest and to evaluate parameters of cardiac function including Ejection Fraction (EF) and Fractional Shortening (FS).

#### Spirometry

Pulmonary function assessment was performed by spirometry using a BTL-08 Spiro system (BTL Industries Limited). The following parameters were measured: Forced Vital Capacity (FVC) and Forced Expiratory Volume in the first second (FEV1).

#### Step or Arm Movement Count by Wristband Activity Tracker

Both ambulatory and non-ambulatory patients were equipped with a Garmin Vívosmart 4 wristband activity tracker for the continuous monitoring of daily step count for ambulatory patients or arm movements count for non-ambulatory patients. The collected monthly recordings underwent statistical analysis. Records with a gap exceeding 6 h were excluded from the analysis.

#### Comparative Analysis of Efficacy Outcomes Assessed in the Upper Extremities of Three DMD Patients by EMG and Functional Tests Up to 12 months After Intraosseous Administration of DT-DEC01 Therapy

The comparative analysis of efficacy outcomes was assessed in the upper extremities of all three DMD patients, regardless of the ambulatory status. Therefore, the correlation was assessed between MUP duration values recorded by EMG in the selected muscles of the upper extremity (the deltoideus and the biceps brachii) and the upper extremity functional tests of the Performance of Upper Limb (PUL 2.0) and the grip strength test measured by the dynamometer at the baseline and at 3, 6, and 12 months after DT-DEC01 therapy administration.

### Statistics

The analysis for statistical significance was performed using GraphPad Prism ver. 10.0 software. Preliminary efficacy outcomes were assessed at the subsequent follow-up visits after systemic administration of the single dose of DT-DEC01 therapy and were compared to the baseline values recorded at screening visit (V0a). Data from: the hand grip strength assessment, the duration of the MUP in EMG, and step or arm movement count are shown as mean ± SEM. The normality of the data was verified by the Shapiro–Wilk test. Parametric two-way ANOVA with Tukey's post-hoc test was used for normally distributed data. For data with an asymmetric distribution, the non-parametric Kruskal–Wallis test with Dunn’s multiple comparisons was applied. P values were considered significant below 0.05.

## Results

### Study Population

Currently, three DMD patients completed a minimum of 12-month follow-up after intraosseous administration of a single, low dose (2 × 10^6^ cells per kg) of DT-DEC01 therapy. Each DT-DEC01 batch represented a personalized product containing DEC cells derived from a PEG-mediated ex vivo fusion of the patient's autologous myoblasts with normal allogeneic myoblasts from the respective donor, the patient's father (Fig. 2). Patients' characteristics including demographic data were previously reported [35].

Patient 1 (ambulatory) a 6-year-old at screening, with genetically confirmed DMD (exon 3–12 deletion). Patient received steroid therapy for 6 months before inclusion in the study. As of 8/15/2023, the patient is 8 years old and has completed 627 days (21 months) of follow-up after DT-DEC01 therapy administration.

Patient 2 (non-ambulatory) a 15-year-old at screening, with genetically confirmed DMD (exon 48–50 deletion). Patient has been wheelchair-dependent since the age of 11 years and 10 months. Patient received steroid therapy for 11 years before inclusion in the study. As of 8/15/2023, the patient is 16 years old and has completed 552 days (18 months) of follow-up after DT-DEC01 therapy administration.

Patient 3 (ambulatory) a 6-year-old at screening, with genetically confirmed DMD (nonsense mutation) Patient received steroid therapy for 2 years before inclusion in the study. As of 8/15/2023, the patient is 7 years old and has completed 515 days (17 months) of follow-up after DT-DEC01 therapy administration.

According to the study protocol, all patients will continue the standard steroid therapy throughout the entire duration of the study.

The Study Flow Diagram with patient disposition is outlined in Fig. 1A.

### Clinical Outcomes

#### Safety and Immunogenicity Outcomes

##### Confirmation of DT-DEC01 Therapy Safety Up to 21 months After Systemic-Intraosseous Administration

Safety of DT-DEC01 therapy was a primary outcome measure of this first-in-human study. Therefore, the patients were continuously monitored for any potential side effects following intraosseous administration of the single dose of 2 × 10^6^ DEC cells/kg body weight. After the first 17 (patient 3) — 21 (patient 1) months following DT-DEC01 therapy administration, none of the patients experienced any therapy related adverse events. There were no reports on Adverse Events of Special Interest (AESI), such as surgical site inflammation, tenderness, fever, nausea, or fatigue. As of 8/15/2023 no study-related Adverse Events (AEs) or Serious Adverse Events (SAEs) were reported throughout an average period of 564 days (19 months) following DT-DEC01 administration. To further investigate the potential immunogenicity of DT-DEC01 therapy, serum samples from all three patients were tested for anti-HLA antibodies at baseline, prior to DT-DEC01 administration, and during follow-up visits at 1, 3-, 6-, 12-, and 18-months post-transplantation. At all-time points, none of the patients exhibited the presence of donor-specific antibodies (DSA), indicating that the transplanted DEC cells were well tolerated without eliciting an immune response.

### Functional Outcomes

#### Preliminary Efficacy Outcomes up to 12 Months After Intraosseous Administration of DT-DEC01 Therapy

##### Patient 1 (Ambulatory)

At 6-month mid-term visit (V6) functional tests revealed 6MWT improvement of 9.1%, with an increase of 39 m in distance [35], whereas at 12-month’s visit V7 the performance returned to baseline level (Table 1). Timed tests of the North Star Ambulatory Assessment (NSAA) demonstrated a 12.8% improvement in the time to stand from supine at 12-month visit (V7), while the 10-m walk/run time returned to baseline level (Table 1).

**Table 1:** Functional tests outcomes assessed over a 12-month study follow-up period after systemic‑intraosseous administration of DT-DEC01 therapy in ambulatory Patient 1

Parameter		Baseline (V0a)	Month 1 (V4)	Month 3 (V5)	Month 6 (V6)	Month 12 (V7)	Change from baseline at Month 12
6MWT (m)		430	427	453	469	415	−3.49%
NSAA	Supine to stand (s)	6.35	5.20	6.87	8.27	5.54	−12.75%
	10 m walk/run (s)	4.99	4.69	5.43	4.29	4.90	−1.80%
Grip strength	Right hand (kg)	5.00 ± 1.56	5.77 ± 0.49	6.53 ± 0.18	6.60 ± 0.51	6.70 ± 0.21	34.00%
	Left hand (kg)	4.00 ± 0.58	5.93 ± 0.32	6.00 ± 0.27	5.60 ± 0.21	6.70 ± 0.31**	67.50%**
EMG: the average MUP duration (ms)	Deltoideus	3.65 ± 0.21		4.84 ± 0.29**	4.18 ± 0.12	4.55 ± 0.30	24.5 ± 8.2%
	Biceps brachii	3.96 ± 0.14		5.83 ± 0.26*	7.08 ± 0.76*	3.86 ± 0.11	−2.5 ± 2.8%
	Rectus femoris	3.27 ± 0.19		3.73 ± 0.14	4.59 ± 0.40*	3.63 ± 0.18	11.0 ± 5.4%
	Gastrocnemius	4.37 ± 0.25		3.47 ± 0.29	5.61 ± 0.58	6.66 ± 0.17	52.6 ± 4.0%

Grip strength data expressed as mean ± SEM (*n* = 3); statistical significance assessed by two-way ANOVA in comparison to the baseline result; EMG data expressed as mean ± SEM (*n* ≥ 4); statistical significance assessed by Kruskal–Wallis test. * *P* ≤ 0.05,** *P* ≤ 0.01

**Fig. 3:**
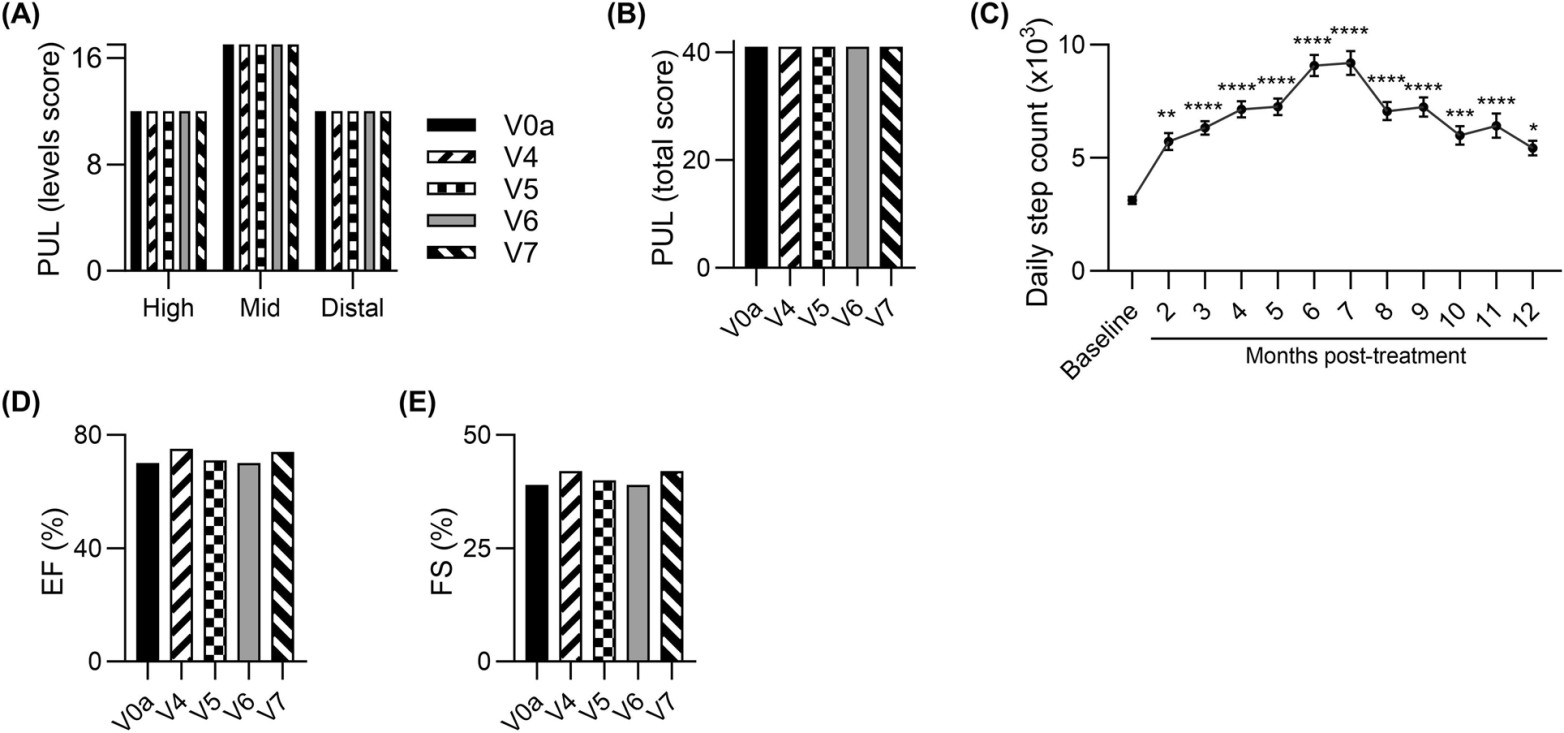
Functional tests outcomes assessed over a 12- month study follow-up period after systemic‑intraosseous administration of DT-DEC01 therapy in ambulatory Patient 1. **A**, **B** Assessment of the PUL 2.0 revealed preservation of the upper limb performance over the entire 12-month follow-up, including maintenance of (**A**) three domains of the upper limb score and (**B**) the total PUL 2.0 score. **C** The assessment of average daily step count recorded by a wristband activity tracker in ambulatory Patient 1 up to 12 months after systemic‑intraosseous DT-DEC01 administration, revealed a significant increase over the entire 12-month follow-up period. **D**, **E** Echocardiography assessment of (**D**) Ejection fraction (EF) and (**E**) Fractional shortening (FS) revealed improvement of both EF by 4% and FS by 8%, when compared to the baseline. Data expressed as mean ± SEM; statistical significance assessed by Kruskal–Wallis test, * *P* ≤ 0.05, ** *P* ≤ 0.01, *** *P* ≤ 0.001, **** *P* ≤ 0.0001. Abbreviations: EF—Ejection Fraction, FS—Fractional Shortening, PUL—Performance of Upper Limb, V0a – screening visit, V4 – 1-month, V5 – 3-months, V6 – 6-months, and V7 – 12-months visit after DT-DEC01 administration

Furthermore, over the 12-month follow-up the patient maintained his upper limb function assessed by PUL 2.0 test, at all three levels (high, mid and distal) (Fig. 3A) and PUL total score (Fig. 3B). The skeletal muscle function assessed at 12-month visit by standard hand grip strength measurement with dynamometer revealed improvement in the right hand by 34.0% (from 5.0 kg at the baseline to 6.7 kg at 12 months post-transplant) and in the left hand by 67.5% (*P* ≤ 0.01) (from 4.0 kg to 6.7 kg) (Table 1).

Recordings of steps count by Garmin Vívosmart 4 increased from an average of 3120 ± 163 steps per day (the weeks 3–5 following the DT-DEC01 administration when recordings started) to an average of 5438 ± 321 steps per day at 12 months, with peak values at months 6 and 7 post-treatment (with an average of 9084 ± 459 steps per day and 9195 ± 528 steps per day, respectively). Step count recordings collected with wristband activity tracker showed variability, but the overall outcome at the 12-month time point, revealed a significant improvement of 74.3% (*P* ≤ 0.05) compared to baseline (Fig. 3C).

To further analyze the restoration of skeletal muscle activity and function, Electromyography (EMG) was performed at baseline and at 3-, 6- and 12-month visits. At 12 months post-transplant (V7), improvements were observed by increase in the average duration of motor unit potentials (MUP) in the deltoideus muscle by 24.5%, in the rectus femoris muscle by 11.0%, and in the gastrocnemius muscle by 52.6%, whereas biceps MUP values were comparable with baseline (Table 1).

Echocardiography assessment of Ejection fraction (EF) and Fractional shortening (FS) revealed improvements of 5.7% and 7.7%, respectively (Fig. 3D and E).

##### Patient 2 (Non-ambulatory)

**Fig. 4:**
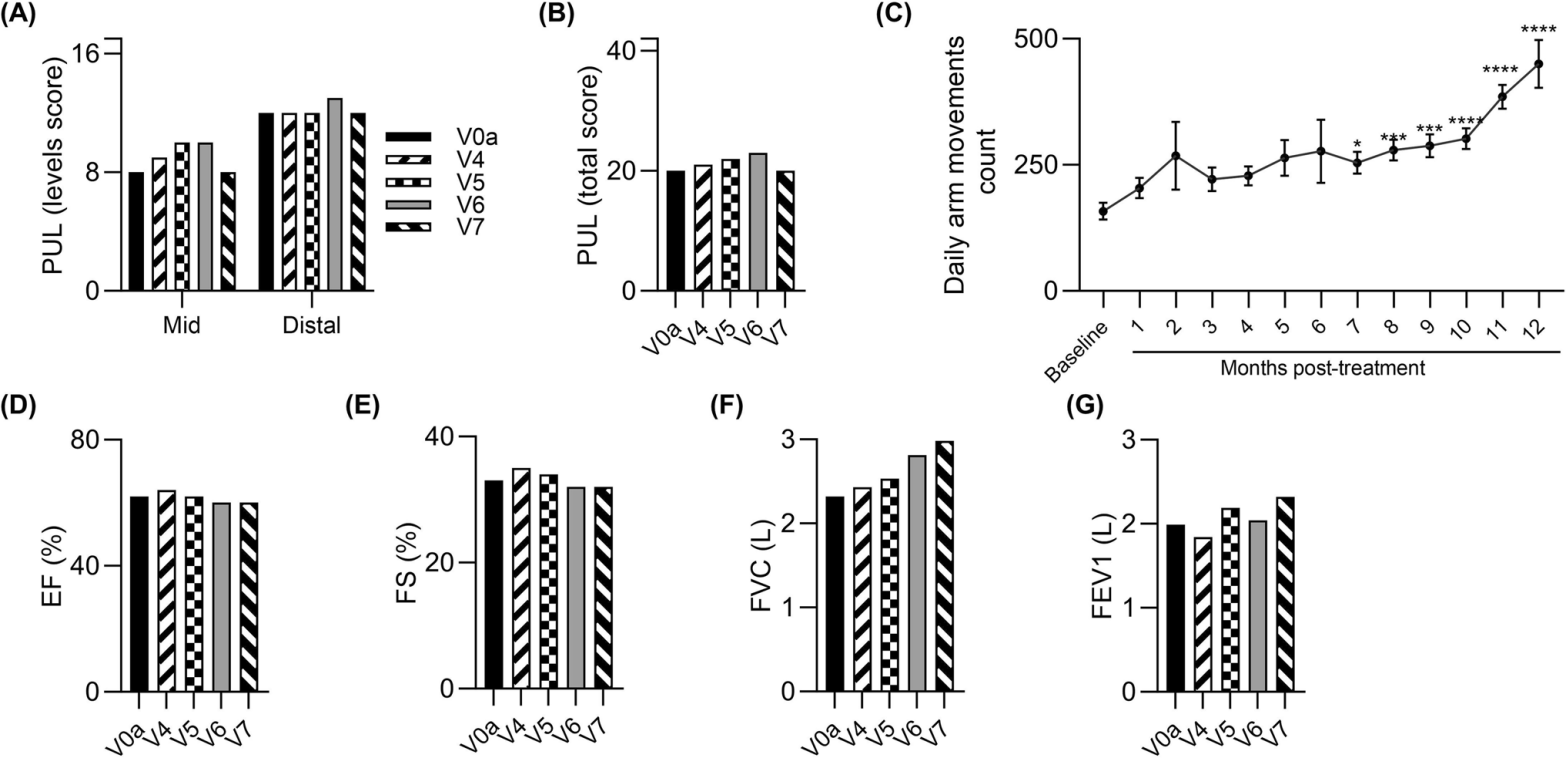
Functional tests outcomes assessed over a 12- month study follow-up period after systemic‑intraosseous administration of DT-DEC01 therapy in non-ambulatory Patient 2. **A**, **B** Assessment of the PUL 2.0 revealed preservation of the upper limb performance over the entire 12-month follow-up, including maintenance of (**A**) two domains of the upper limb score and (**B**) the total PUL 2.0 score. **C** The assessment of average arm movements count recorded with a wristband activity tracker in non-ambulatory Patient 2 up to 12 months after systemic‑intraosseous DT-DEC01 administration revealed an increase over the entire 12-month follow-up period. The differences in the count of arm movements were significant from month 7 up to month 12 following DT-DEC01 administration. **D**, **E** Echocardiography assessment of (**D**) Ejection fraction (EF) and (**E**) Fractional shortening (FS) revealed maintenance at baseline level. **F**, **G** Spirometry assessment at 12-months visit (V7) revealed improvement in the respiratory parameters of (**F**) FVC by 29% and (**G**) FEV1 by 17% compared to baseline. Data expressed as mean ± SEM; statistical significance assessed by Kruskal–Wallis test, * *P* ≤ 0.05, ** *P* ≤ 0.01, *** *P* ≤ 0.001, **** *P* ≤ 0.0001. Abbreviations: EF—Ejection Fraction, FEV1—Forced Expiratory Volume in the first second, FS—Fractional Shortening, FVC—Forced Vital Capacity, PUL—Performance of Upper Limb, V0a – screening visit, V4 – 1-month, V5 – 3-months, V6 – 6-months, and V7 – 12-months visit after DT-DEC01 administration

The preliminary efficacy outcomes assessed at 12-month follow-up, revealed gradual improvement in PUL 2.0 entry score from 0 points with no useful hand activity at screening visit (V0a) to 2 points with ability to rise hands to mouth at 1-month visit (V4) and 3 points allowing to raise a loaded cup to the mouth at 3- and 6-month visits (V5 and V6) [35]. At 12-months (V7) visit his entry score decreased to 1 point with hand function allowing to hold a pen, pick up a coin or drive a powered chair, still showing improvement compared to baseline score of 0 before DT-DEC01 administration. These observations were consistent with improvements in mid- and distal level scores and PUL total score observed up to 6 months (V6) [35], followed by a return to baseline values recorded at the 12-month visit (V7) after DT-DEC01 administration (Fig. 4A and B). Similarly, assessment of grip strength revealed a significant increase in both, right and left hand observed up to 6 months (V6) [35], followed by a decrease, however at 12-months’ time point (V7) the grip strength in both hands was still increased by 12.5% in the right-, and by 11.6% in the left hand, compared to baseline before DT-DEC01 administration (Table 2).

**Table 2:** Functional tests outcomes assessed over a 12- month study follow-up period after systemic‑intraosseous administration of DT-DEC01 therapy in non-ambulatory Patient 2

Parameter		Baseline (V0a)	Month 1 (V4)	Month 3 (V5)	Month 6 (V6)	Month 12 (V7)	Change from baseline at Month 12
Grip strength	Right hand (kg)	8.00 ± 0.00	8.28 ± 0.15	10.03 ± 0.43***	9.767 ± 0.29**	9.00 ± 0.40	12.50%
	Left hand (kg)	8.33 ± 0.33	9.90 ± 0.06**	10.20 ± 0.27**	10.50 ± 0.15***	9.30 ± 0.35	11.64%
EMG: the average MUP duration (ms)	Deltoideus	2.60 ± 0.23		5.66 ± 0.46****	4.96 ± 0.40	3.48 ± 0.24	33.6 ± 9.3%
	Biceps brachii	2.12 ± 0.14		3.86 ± 0.17	5.15 ± 0.28****	5.29 ± 0.41****	149.6 ± 19.3%****
	Rectus femoris	3.16 ± 0.12		3.27 ± 0.12	3.11 ± 0.15	5.16 ± 0.25**	63.5 ± 7.8%**
	Gastrocnemius	5.87 ± 0.27		4.72 ± 0.16	7.62 ± 0.28	7.82 ± 0.45	33.2 ± 7.6%

Grip strength data expressed as mean ± SEM (*n* = 3); statistical significance assessed by two-way ANOVA in comparison to the baseline result; EMG data expressed as mean ± SEM (*n* ≥ 10); statistical significance assessed by Kruskal–Wallis test. ** *P* ≤ 0.01, *** *P* ≤ 0.001, **** *P* ≤ 0.0001

Patient average arm movement counts recorded by Garmin Vivosmart 4 showed continuous increase after DT-DEC01 administration from 158 ± 17 arm movements daily at baseline to 450 ± 47 at 12 months after DT-DEC01 therapy administration, revealing significant improvement by 184.6% (*P* ≤ 0.0001) (Fig. 4C).

To further analyze the restoration of skeletal muscle activity and function, EMG was performed at baseline and at V5, V6, and V7 visit. At 12 months post-transplant (V7), EMG revealed increase in the average MUP duration in all tested muscles of upper and lower extremity: in deltoideus muscle by 33.6%, in biceps brachii muscle by 149.6% (*P* ≤ 0.0001), in rectus femoris muscle by 63.5% (*P* ≤ 0.01) and in gastrocnemius muscle by 33.2% (Table 2).

Moreover, the EF and FS parameters values assessed by ECHO were maintained and comparable to the baseline values over the entire 12-month follow-up period (Fig. 4D and E).

Spirometry assessment at 12-month visit (V7) revealed improvement in the respiratory parameters of FVC by 28.5% and FEV1 by 16.6% (Fig. 4F and G).

##### Patient 3 (Ambulatory)

The preliminary efficacy outcomes assessed at 12-month visit (V7), revealed improvement in 6MWT by 17.4% and increase in 6MWD by 59 m (Table 3). The timed functions of NSAA test demonstrated an improvement in time to stand from supine by 27.4% (time shorter by 2.1 s), and in 10-m walk/run time by 11.6% (time shorter by 0.5 s) (Table 3).

**Table 3:** Functional tests outcomes assessed over a 12- month study follow-up period after systemic‑intraosseous administration of DT-DEC01 therapy in ambulatory Patient 3

Parameter		Baseline (V0a)	Month 1 (V4)	Month 3 (V5)	Month 6 (V6)	Month 12 (V7)	Change from baseline at Month 12
6MWT (m)		339	334	345	390	398	17.40%
NSAA	Supine to stand (s)	7.48	5.72	6.19	7.08	5.43	−27.41%
	10 m walk/run (s)	4.41	4.60	5.30	4.72	3.90	−11.56%
Grip strength	Right hand (kg)	2.88 ± 0.11	2.03 ± 0.22	2.53 ± 0.34	3.07 ± 0.20	2.90 ± 0.10	0.67%
	Left hand (kg)	2.51 ± 0.21	2.07 ± 0.30	1.93 ± 0.23	2.17 ± 0.18	3.90 ± 0.40	55.38%**
EMG: the average MUP duration (ms)	Deltoideus	3.76 ± 0.33		6.21 ± 0.49**	5.94 ± 0.34*	6.01 ± 0.29**	59.9 ± 7.6%**
	Biceps brachii	3.49 ± 0.36		4.17 ± 0.30	5.10 ± 0.18**	4.60 ± 0.19	31.6 ± 5.5%
	Rectus femoris	4.06 ± 0.34		6.33 ± 0.45*	4.83 ± 0.27	4.52 ± 0.18	11.3 ± 4.4%
	Gastrocnemius	4.92 ± 0.16		6.09 ± 0.22	7.15 ± 0.34***	5.47 ± 0.27	11.0 ± 5.5%

Grip strength data expressed as mean ± SEM (*n* = 3); statistical significance assessed by two-way ANOVA in comparison to the baseline result; EMG data expressed as mean ± SEM (*n* ≥ 10); statistical significance assessed by Kruskal–Wallis test. ** *P* ≤ 0.05, ** *P* ≤ 0.01, *** *P* ≤ 0.001

**Fig. 5:**
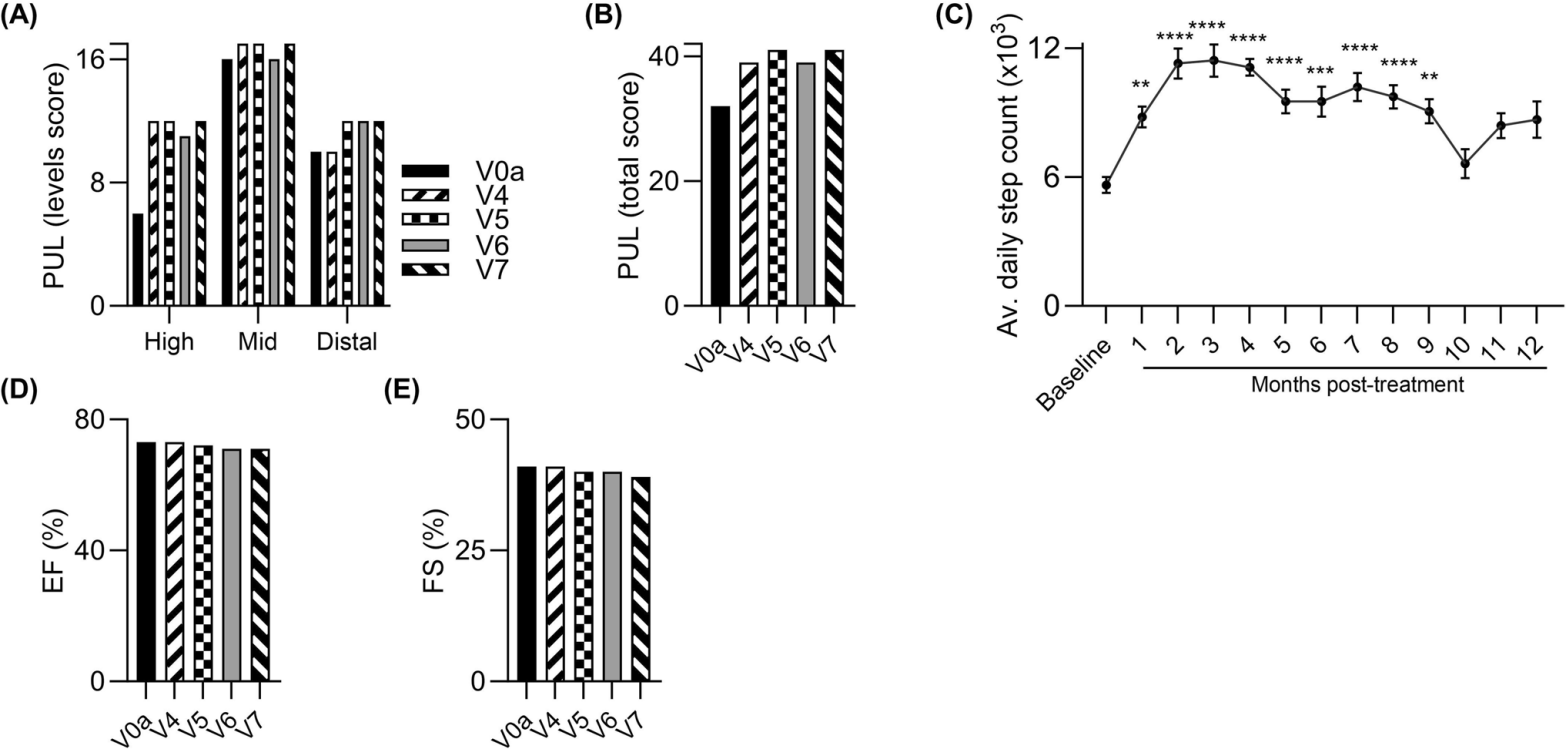
Functional tests outcomes assessed over a 12-month study follow-up period after systemic‑intraosseous administration of DT-DEC01 therapy in ambulatory Patient 3. **A**, **B** Assessment of the PUL 2.0 revealed improvement of the upper limb performance over the entire 12-month follow-up, including improvements in (**A**) three domains of the upper limb score and (**B**) the total PUL 2.0 score. **C** The assessment of average daily step count recorded with a wristband activity tracker in ambulatory Patient 3 up to 12 months after systemic‑intraosseous DT-DEC01 administration revealed an increase over the entire 12-month follow-up period. The differences in the step count were significant from month 1 up to month 9 following DT-DEC01 administration. **D**, **E** Echocardiography assessment of (**D**) Ejection fraction (EF) and (**E**) Fractional shortening (FS) revealed maintenance of both EF and FS over the entire 12-month follow-up. Data expressed as mean ± SEM; statistical significance assessed by Kruskal–Wallis test, * *P* ≤ 0.05, ** *P* ≤ 0.01, *** *P* ≤ 0.001, **** *P* ≤ 0.0001. Abbreviations: EF—Ejection Fraction, FS—Fractional Shortening, PUL—Performance of Upper Limb, V0a – screening visit, V4 – 1-month, V5 – 3-months, V6 – 6-months, and V7 – 12-months visit after DT-DEC01 administration

Furthermore, over the 12-month follow-up the patient improved his upper limb function assessed by PUL 2.0 test, at all three levels (high, mid and distal) (Fig. 5A) and in PUL total score (Fig. 5B). The skeletal muscle function assessed at V7 by standard hand grip strength measurements with dynamometer, revealed maintenance of strength in the right hand at baseline level and 55.4% (*P* ≤ 0.01) increase in the strength of left hand (from 2.5 kg to 3.9 kg), (Table 3).

Recordings of steps count by Garmin Vívosmart 4 increased from an average of 5634 ± 363 steps per day at baseline to an average of 8683 ± 846 steps per day at 12-month visit revealing improvement of 54.1%, whereas a peak value of an average of 11 420 ± 756 steps per day was recorded at 3 months after DT-DEC01 therapy administration (Fig. 5C).

To further analyze the restoration of skeletal muscle activity and function, EMG was performed at the baseline and at 3-, 6- and 12-month visits. At 12 months post-transplant (V7), improvements were recorded in all selected muscles of upper and lower extremity by increase in the average duration of MUP: in the deltoideus muscle by 59.9% (*P* ≤ 0.01), in the biceps brachii muscle by 31.6%, in the rectus femoris muscle by 11.3% and in gastrocnemius muscle by 11.0% (Table 3).

The EF and FS parameters values assessed by ECHO were comparable to the baseline over the entire 12-month follow-up (Fig. 5D and E).

##### Confirmation of Correlation between Improved EMG Parameters and Functional Tests Assessed in the Upper Extremities of Three DMD Patients Up to 12 months After Intraosseous Administration of DT-DEC01 Therapy

**Fig. 6:**
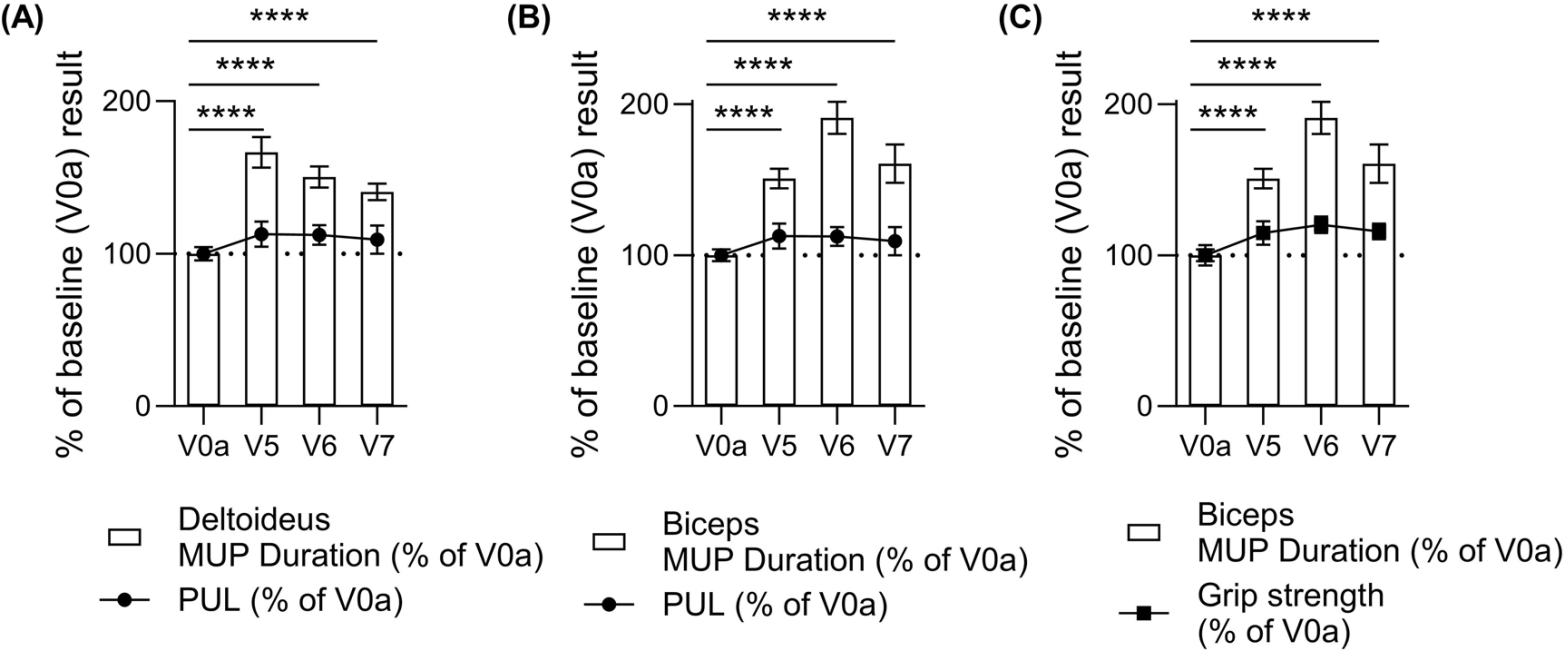
Comparative analysis of Motor Unit Potentials (MUP) duration, assessed by EMG in the deltoideus and the biceps brachii muscles, and upper extremity functional tests of PUL 2.0 and grip strength assessed in three DMD patients from baseline up to 12 months after systemic-intraosseous administration of the DT-DEC01 therapy. Comparative analysis of MUP duration and upper extremity functional tests revealed: **A** Correlation between increase in MUP duration recorded in the deltoideus muscle and improvement of functional outcomes assessed by the PUL test, **B** Correlation between increase in MUP duration recorded in the biceps brachii muscle and improvement of functional outcomes assessed by the PUL test and **C** Correlation between increase in MUP duration recorded in the biceps brachii muscle and improvement of functional outcomes assessed by grip strength over the 12-month follow-up period after DT-DEC01 therapy administration. Data expressed as mean ± SEM; statistical significance of MUP duration data assessed by Kruskal–Wallis test, * *P* ≤ 0.05, ** *P* ≤ 0.01, *** *P* ≤ 0.001, **** *P* ≤ 0.0001. Abbreviations: MUP—Motor Unit Potentials, V0a – screening visit, V5—3-months, V6 – 6-months, V7 – 12‑months visit after DT-DEC01 administration

At 12 months after DT-DEC01 administration, when compared to the baseline, the EMG assessments of selected muscles of the upper extremity (deltoideus and biceps brachii) in three DMD patients revealed 40.5% increase in MUP duration recorded in the deltoideus muscle which correlated with improved functional outcomes assessed by the PUL 2.0 test (by 9.3%) (Fig. 6A). Furthermore, an increase in MUP duration recorded in the biceps brachii muscle (by 60.5%) correlated with improved functional outcomes of upper extremity assessed by the PUL 2.0 test (Fig. 6B). Moreover, increase in MUP duration in the biceps brachii correlated with 15.7% increase in grip strength measured by dynamometer (Fig. 6C).

## Discussion

The hallmark of DMD represents constant muscle degeneration and regeneration, leading to progressive muscle weakness, inflammation, and fibrosis of the skeletal, cardiac, and respiratory muscles. This corresponds with the decline of ambulatory function and cardiac and respiratory failure, which have not been halted or slowed down over the past years, regardless of the introduction of new generations of supportive measures and therapeutic approaches [41–45]. Therefore, despite the efforts, there is no cure for DMD patients.

The ultimate goal of therapeutic options for DMD is to introduce safer, efficacious and universal therapies applicable to all DMD patients regardless of genetic mutation, age or ambulatory status.

To meet these goals, we have developed a novel, Dystrophin Expressing Chimeric (DEC) cell therapy generated by ex-vivo fusion of human myoblasts from normal and DMD-affected donors [28–34]. DEC cells were extensively tested in preclinical studies where long-term safety and engraftment correlated with dystrophin expression and functional improvements in the DMD-affected organs including cardiac, respiratory and skeletal muscles. Moreover, there was no need for immunosuppression [28, 30].

In preparation for clinical application of DEC cells, we have established a clinical and manufacturing protocol for development of personalized DT-DEC01 cell therapy representing the Advanced Therapy Medicinal Product Product (ATMP).

Recently, we have reported interim results of the first-in-human pilot study, on the safety of the systemic intraosseous DT-DEC01 administration, confirmed by the absence of Adverse Events (AE), Severe Adverse Events (SAE) or evidence of any immune responses, confirmed by the lack of presence of anti-HLA antibodies over the 6-month follow-up period. Furthermore, the efficacy of DT-DEC01 therapy was confirmed by a consistent improvement in standard and objective functional tests for up to six months after DT-DEC01 administration [35].

In the current study, we aimed to further investigate the safety and efficacy of DT-DEC01 therapy for the extended period of time up to 21 months after systemic-intraosseous administration. Our findings provide additional confirmation of DT-DEC01 therapy safety, by lack of any reports of treatment-related Adverse Events (AE) or Serious Adverse Events (SAE). Moreover, the absence of anti-HLA antibodies in all three patients further supports the safety and tolerability of DT-DEC01 therapy.

Furthermore, the preliminary efficacy outcomes assessed at 12 months after DT-DEC01 administration, confirmed continuous improvements in some functional tests and/or maintenance of some parameters at the baseline level.

Improvements were found in ambulatory patients, during the first 6 months after DT-DEC01 therapy [35] as evidenced by the 6-min walk test (6MWT). Subsequently, follow-up visits either showed continuous improvement or maintenance at the baseline at the 12-months visit. Furthermore, functional improvements in timed functions of NSAA (supine to stand and 10 m walk/run test) were confirmed at the 12- months visit for ambulatory patients. Interestingly, the PUL 2.0 test in both younger ambulatory patients revealed maintenance of the entry score during the entire 12-month follow up period. In contrast, the older non-ambulatory patient showed significant improvement, transitioning from no useful hand activity at the screening visit to regaining some hand functions, reaching the peak of performance of raising a loaded cup to the mouth recorded at 3- and 6-month visits. Notably, there was also a significant increase in the grip strength observed over the entire 12-month follow-up period for both ambulatory and non-ambulatory patients.

It is important to emphasize the correlation between improved EMG parameters and functional tests observed in all three DMD patients at 12 months after intraosseous administration of DEC-01 therapy. Specifically, improvements in the upper extremity functional tests of PUL.02 correlated with increased duration of MUP recorded in the deltoideus and the biceps brachii muscles, whereas improvements in grip strength correlated with increased MUP duration recorded by EMG in the biceps brachii muscles. The improvements recorded by the standard functional tests assessed in all three patients both ambulatory and non-ambulatory over the entire 12- months of study duration following systemic- intraosseous DT-DEC01 administration, were consistent with the observations of patients' daily activities such as step count for ambulatory patients and arm movements count for non-ambulatory patients recorded by the wristband activity tracker. Remarkably, all three patients demonstrated a significant increase in their daily activities ranging from 54.1% to 184.6% over the 12 months period following DT-DEC01 therapy administration, providing further evidence of the positive effect of DT-DEC01 on the overall functioning of DMD patients.

The preliminary efficacy of systemic intraosseous administration of DT-DEC01 was also confirmed by Echocardiography assessment, showing either improvement or maintenance of EF and FS values at the baseline level over the entire 12- months of study follow-up. It is worth mentioning that maintenance of EF and FS at baseline values in the older, 16 years old non-ambulatory DMD patient is an important finding, considering the expected progression of the cardiac function decline at this stage of DMD disease [46–54].

Furthermore, another notable observation, confirming the systemic response to the intraosseous DT-DEC01 therapy administration was recorded by the spirometry test in the older non-ambulatory patient at 12 months visit, where FVC values showed a significant 29% increase, and FEV1 increased by 17% over baseline values assessed at the screening visit. This is again a significant finding considering the progressive impairment of pulmonary function observed in older non-ambulatory DMD patients [55–58].

There are several benefits of DT-DEC01 therapy which have to be emphasized. First, our study has provided compelling evidence of DT-DEC01 therapy safety confirmed by absence of the therapy related AE and SAE. Moreover, it does not trigger an immune response after systemic-intraosseous administration, as demonstrated by negative results of anti-HLA antibodies. This finding confirms the significant advantage of DT-DEC01 therapy, since it eliminates the need for immunosuppression, which is associated with serious side effects and complications. Furthermore, compared to other approaches tested for DMD [12, 13, 17, 59] DT-DEC01 therapy does not require genetic manipulation therefore eliminates the risk of off-target mutations. Moreover, the creation of DT-DEC01 does not rely on viral vectors for delivery, reducing the risk of sensitization, thus allowing for readministration if desired. The most unique feature of DT-DEC01 therapy is that it is not dependent on the genetic mutation of the DMD patient; therefore, it may be considered for all DMD patients. Our preliminary safety and efficacy data confirm improvements and/or lack of disease progression recorded by standard functional tests, EMG, ECHO and spirometry in both younger ambulatory patients and the older non-ambulatory boy, providing evidence that DT-DEC01 therapy can be applied to all DMD patients regardless of gene mutation, age and ambulatory status. These characteristics position DT-DEC01 as a universal therapy for all DMD patients, and may be considered for patients with other muscular dystrophies.

The ability to target a broad range of patients with muscular dystrophies without the need for genetic modification and the fear of sensitization significantly expands therapeutic potential and applicability of DT-DEC01 therapy.

This study has some limitations that should be mentioned. First, this study reports preliminary safety and efficacy outcomes based on the assessment of three DMD patients enrolled in the protocol. Assessing a small cohort of patients over a longer follow-up period can indeed be challenging, especially in the context of a rare disease like DMD, where there may be limited access to patients who meet the specific inclusion criteria for the study.

However, despite these challenges the presented data is promising, showing either continuous improvements in some functional parameters or maintenance of others at the baseline level assessed over the 12-month follow-up after systemic-intraosseous administration of DT-DEC01 therapy. Specifically, the maintenance of cardiac parameters of EF and FS at the baseline level and improvement of pulmonary function confirmed by significantly increased FVC and FEV1 values recorded by spirometry in the older non-ambulatory patient indicates both, the systemic effect of intraosseous administration of DT-DEC01 and the potential to halt progression of DMD. These outcomes are encouraging considering the progressive nature of DMD, however additional data from these three patients and others being included in the study will provide further justification of the assessed functional outcomes.

In our preclinical studies we have confirmed long-term engraftment and biodistribution of human DEC cells specifically to the DMD-targeted organs of heart, diaphragm and skeletal muscles which corresponded with dystrophin expression and significant improvement of function [33, 34].

However, in our clinical study, we have not taken muscle biopsies for dystrophin assessment by immunoblots due to the safety concerns of performing muscle biopsies in the pediatric population under anesthesia and exposing them to anesthesia-related complications to which DMD patients are prone [60–68]. Additionally, there is a debate on the validity of Western Blot results and their correlation with functional outcomes as raised by many investigators and recent FDA reports [9–12, 14, 15, 69–75]. Therefore, we have chosen a less invasive electromyography (EMG) assessments of muscle function after DT-DEC01 administration and confirmed the role of EMG as a reliable biomarker for monitoring and recording changes in muscle activity correlating with functional improvements assessed by standard tests [35, 76–82].

In summary, when compared with our interim report at six months after DT-DEC01 therapy [35] the current study confirmed safety and efficacy of DT-DEC01 therapy up to 21 months after intraosseous administration.

Over the 12-month follow-up period, there was continuation of improvements in some functional parameters, including the 6MWT and NSAA timed function tests (time-to-stand and time to run or walk 10 m) in ambulatory patients. Additionally, there were improvements in the PUL test and grip strength, which correlated with increased MUP duration assessed by EMG in both ambulatory and non-ambulatory patients. Notably, some parameters that showed a peak of performance at 6 months [35] returned to baseline values between 6 and 12 months following DT-DEC01 administration. These findings are important to consider for future clinical trials involving DT-DEC01 therapy redosing.

This study supports the potential benefits of DT-DEC01 therapy in improving cardiac, respiratory and skeletal muscle function in DMD patients after systemic-intraosseous administration, providing hope for better outcomes and enhanced quality of life for DMD patients. Additionally, this study also highlights use of EMG as a valuable biomarker for monitoring functional changes in muscles affected by DMD after DT-DEC01 therapy. However, further studies on a larger group of patients are needed to confirm the long-term safety and benefits of DT-DEC01 therapy beyond the 12-month follow-up period.

## Conclusions

This study provides strong evidence supporting the safety and preliminary efficacy of DT-DEC01 therapy for treatment of DMD. The lack of study- related Adverse Events (AE) or Serious Adverse Events (SAE) observed over the 21-month follow-up period confirms the safety of systemic- intraosseous DT-DEC01 administration. The absence of immune response, as indicated by the lack of presence of anti-HLA and DSA antibodies, further supports the therapy’s safety. The administration of a single dose of DT-DEC01 demonstrated some significant functional benefits. Over the course of multiple visits over a 12-month period, ambulatory patients showed improvements in the 6MWT and timed function tests of NSAA, while both ambulatory and non-ambulatory patients exhibited enhancements in PUL 2.0, grip strength, and EMG assessments of MUP duration. Daily activity also improved in all patients, with ambulatory patients showing an increased step count and non-ambulatory patients displaying increased arm movement count for up to 12-months after DT-DEC01 administration. Moreover, the systemic effect of intraosseous DT-DEC01 administration was evident by improved cardiac and pulmonary parameters assessed by ECHO and spirometry. Importantly, these benefits were observed in both ambulatory and non-ambulatory patients, regardless of gene mutation suggesting that DT-DEC01 could serve as a universal therapy for all DMD patients.

In conclusion, this study introduces an innovative concept of personalized myoblast-based cellular therapy and establishes DT-DEC01 as a promising and universally effective treatment option for DMD patients. The findings from this study provide a strong basis for future clinical trials and further support the potential of DT-DEC01 therapy in significantly improving the lives of individuals affected by Duchenne muscular dystrophy.

## Data Availability

All data generated or analyzed during this study are included in this published article.

## Abbreviations

6MWT: 6-Minute Walk Test
6MWD: 6-Minute Walk Distance
AE: Adverse Event
AESI: Adverse Event of Special Interest
ANOVA: Analysis of Variance
DEC: Dystrophin Expressing Chimeric (Cells)
DMD: Duchenne Muscular Dystrophy
DSA: Donor-Specific Antibodies,
ECHO: Echocardiography
EF: Ejection Fraction
EMG: Electromyography
FACS: Fluorescence-Activated Cell Sorting
FEV1: Forced expiratory volume in the first second
FS: Fractional Shortening
FVC: Forced Vital Capacity
GCP: Good Clinical Practice
HLA: Human Leukocyte Antigen
MUP: Motor Unit Potential
NSAA: North Star Ambulatory Assessment
PEG: Polyethylene Glycol
PUL: Performance of Upper Limb
SAE: Serious Adverse Event
